# Anti-proliferative action of vitamin D in MCF7 is still active after siRNA-VDR knock-down

**DOI:** 10.1186/1471-2164-10-499

**Published:** 2009-10-28

**Authors:** José L Costa, Paul P Eijk, Mark A van de Wiel, Derk ten Berge, Fernando Schmitt, Carmen J Narvaez, JoEllen Welsh, Bauke Ylstra

**Affiliations:** 1 Department of Pathology, VU University Medical Center (VUMC), Amsterdam, The Netherlands; 2 Institute of Molecular Pathology and Immunology of the University of Porto - IPATIMUP, University of Porto, Portugal; 3 Department of Epidemiology and Biostatistics, VU University Medical Center (VUMC), Amsterdam, The Netherlands; 4 Erasmus Stem Cell Institute, Department of Cell Biology, Erasmus MC, Rotterdam, The Netherlands; 5 Medical Faculty of Porto University, Porto, Portugal; 6 GenNYsis Center for Excellence in Cancer Genomics, and Department of Biomedical Sciences, University at Albany, New York, USA; 7 Department of Mathematics, VU University, Amsterdam

## Abstract

**Background:**

The active form of Vitamin D, 1,25-dihydroxyvitamin D_3 _(1,25D), has strong anti-proliferative effects, yet the molecular mechanisms underneath this effect remain unclear. In contrast, the molecular mechanism of 1,25D for the regulation of calcium homeostasis has principally been resolved, demonstrating a pivotal role for the vitamin D receptor (VDR).

**Results:**

We first addressed the question whether the anti-proliferative effects of 1,25D are influenced by VDR. Knockdown of VDR by siRNA did not affect the anti-proliferative effects of 1,25D in MCF7 breast cancer cells. This unanticipated finding led us to take an alternative approach using genome wide screens to study the molecular mechanisms of 1,25D in proliferation. For that purpose, four independently developed and stable 1,25D resistant MCF7 cell lines were analyzed. Array CGH identified a copy number alteration in a region of 13.5 Mb at chromosome 11q13.4-14.1 common to all four 1,25D resistant cell lines. Expression arrays revealed that no single gene was differentially expressed between the sensitive and resistant cells, but multiple membrane receptor signaling pathways were altered in the 1,25D resistant cell lines. Importantly, in the genome wide experiments neither *VDR, CYP24A1 *nor other known vitamin D signaling pathway genes were associated with 1,25D resistance.

**Conclusion:**

In conclusion, siRNA and genome wide studies both suggest that the anti-proliferative effects of 1,25D in MCF7 breast tumor cell lines do not rely on classical Vitamin D pathway *per se*.

## Background

Strong anti-proliferative effects of 1,25-dihydroxyvitamin D_3 _(1,25D) have been demonstrated in a wide spectrum of solid cancers, *in vitro *and in animal models [1]. A link between 1,25D status and cancer has also been demonstrated in epidemiological studies. Together these data lead to a number of clinical trials to test the efficacy of 1,25D or synthetic analogs [2]. Yet, the molecular mechanisms by which the 1,25D pathway exerts its anti-proliferative effects remain unclear. There is much need for detailed knowledge of the molecular mechanism behind the anti-proliferative action of 1,25D, since calcemic side-effects form a major obstacle for the development of 1,25D or derivative drugs. Classically, 1,25D mediates the maintenance of calcium homeostasis through activation of the vitamin D receptor (VDR), a ligand dependent transcription factor. The 1,25D-VDR receptor-ligand complex functions by interacting with vitamin D response elements (VDREs) present in the promoter region of responsive genes. While many known VDRE-containing genes contribute to calcium regulation (e.g. *CYP24A1*, osteocalcin and osteopontin), others are involved in cellular processes like proliferation and apoptosis (e.g. *p21*, c-*fos*, and *Bcl*2) [2-4]. Alternative, non-genomic pathways have been suggested to explain the anti-proliferative action of 1,25D. For example a membrane bound VDR was proposed [5] and other forms of signaling outside the nucleus [6].

In previous years, several MCF7 cell lines have been generated of which proliferation is not affected by 1,25D. Their 1,25D resistance was introduced by long time low dose or increasing dose of 1,25D [7,8]. Notably, even when cultured in the absence of 1,25D these cell lines still persist to proliferate whenever exposed to 1,25D. This makes it reasonable to assume that the alterations that led to the resistance need to be (epi-)genetically fixed (chromosomal copy number alterations [CNAs], point-mutations, methylation). We therefore hypothesized that genome wide screens could potentially identify these (epi-)genetic changes in the absence of Vitamin D. In this study we thus utilized genome wide approaches to identify these stable alterations.

Our understanding that stable changes exist in these cell lines which underlie their response to 1,25D, prompted us to avoid the use of 1,25D. To perform this genomics study in the presence of 1,25D would identify the massive number of 1,25D downstream genes involved in proliferation and cell cycle. Indeed, there are various examples in the literature where 1,25D has been used resulting in the identification of such genes [9-13]. Although causative/driver genes may have been identified in these studies, they would be difficult to pinpoint within the massive amount of proliferative genes. Thus, omission of 1,25D in our genomic screens could potentially identify the causative/driver alterations that cause resistance in our model, whilst omitting the detection of downstream proliferative genes. Prior to these experiments we performed VDR knockdown experiments to study the role of this receptor in 1,25D anti-proliferation effect.

These experiments suggest that the anti-proliferative effects of 1,25D can either be dissociated or only require extremely low levels of the VDR in MCF7. In addition we found that structural changes on chromosome 11q13.4-14.1 may be involved in 1,25D resistance of MCF7.

## Methods

### Biological material

Four MCF7 breast tumor cell lines and their 1,25D resistant counterparts were obtained and designated, MCF7^wt1^-MCF7 VD^R^[7], MCF7^wt2^-MCF7 DR, MCF7^wt2^-MCF7 DRA and MCF7^wt3^-MCF7 D3res [8]. The MCF7 VD^R ^resistant line was obtained by exposure to increasing amounts of 1,25D (0.01-10 μM), whereas MCF7 DR, MCF7 DRA and MCF7 D3res were exposed to maximal concentrations of 100 nM 1,25D for up to 12 months. All cell lines were maintained in Dulbecco's modified Eagle's medium (Invitrogen, Leek, The Netherlands), supplemented with 10% fetal bovine serum (Invitrogen), 2 mM glutamine, 100 U/l penicillin and 100 U/l streptomycin and were cultured at 37°C in a humid atmosphere consisting of 5% CO_2 _and 95% air. Cell proliferation was measured by the 3-(4,5-dimethyl-2-thiazolyl)-2,5-diphenyl-2H-tetrazolium bromide (MTT) assay [14] (Sigma-Aldrich, Sintra, Portugal) in the presence of 1,25D 100 nM or vehicle for 72 h.

### VDR knockdown by siRNA

Knockdown of VDR expression was performed on all three separate parental MCF7 cell lines as biological triplicates using the Silencer^® ^Validated siRNA ID 4010 according to the manufacturer's protocols (Ambion Ltd., Huntingdon, United Kingdom). A 21-nucleotide probe (5'-GGAGUUCAUUCUGACAGAUtt-3'), directed against a sequence in exon 4 of the human VDR gene (accession number NM_001017535), was transfected into all three MCF7 parental cell lines using the siPORT™ Amine Transfection Agent (Ambion). Transfection optimization and efficiency was measured using the KDalert GAPDH Assay Kit (Ambion), which measures GAPDH siRNA-induced gene knockdown at the protein level. The efficiency level obtained was within the levels described and according to the manufacturer. The conditions used for optimal silencing were: 20.000 cells/well in 24-well plates, 20 nM of dsRNA and 2 μl of the transfection agent. A negative siRNA (Ambion) with no significant homology to any known gene sequence was used as a control. Transient transfections were performed for 72 h in the absence/presence of 100 nM 1,25D. After this period, the efficacy of VDR knockdown was assessed by Q-PCR (using the primers described in Table 1) and proliferation by MTT assays.

**Table 1:** List of primers used for Q-PCR experiments

		Primers 5' → 3'	
		
Gene symbol	Accession number [GenBank:]	Forward	Reverse
FAM14A	NM_032036	GCCTCTGTTGGGTCAGTGTTG	TCATCTTCTTTAGCCTCGGGTT
CYP24A1	NM_000782	GACTACCGCAAAGAAGGCTAC	CATCACTTCCCCTGGTTTCATTA
PHLDA1	NM_007350	TGAAGGAGGGCGTGCTGG	GCTGCTTGGGCGGGATAA
DDIT4	NM_019058	GGACGCACTTGTCTTAGCAGTTCTC	CCAGGGCGTTGCTGATGAA
CYFIP2	NM_014376	CATTCCGTATCCACCGTCCAA	GCTGGGTAATGAGTCTGTTCAAGTC
FOXC1	NM_001453	CACTCGGTGCGGGAGATGTT	GAGACTGGCTGGAAGGGAAGG
CRABP2	NM_001878	ACAGGAGGGAGACACTTTCTACATCAA	TCCCATTTCACCAGGCTCTTACA
ST6GALNAC5	NM_030965	GCCACTGGACGGATACCTCG	CTGGGAGCCTTGCCGACT
NUAK1	NM_014840	TGAAGAAGCGAAGCAACAGCG	GAGGGTAAGGCAGGACCAACTA
VDR	NM_000376	AGCGGAAGGCACTATTCACC	CATCATGCCGATGTCCACACA
GAPDH	NM_002046	TGAAGCAGGCGTCGGAGGG	CGTCAAAGGTGGAGGAGTGGGT

### Western blot and antibodies

Protein lysates were made of cells at approximately 70-80% confluency. Cells were washed twice with PBS, lysed on ice [1% (v/v) Triton X-100 and 1% (v/v) NP-40 in desionized PBS with 1:7 proteases inhibitors cocktail], vortexed and clarified by centrifugation at 14000 × rpm for 10 min at 4°C. Protein concentration was determined using Dc protein assay (Bio-Rad, Amadora, Portugal). A total of 50 μg of total protein lysates were analyzed by 8% SDS-polyacrylamide gel electrophoresis and transferred to a Amersham Hybond ECL nitrocellulose membrane (GE Healthcare, Carnaxide, Portugal), using a Trans-Blot SD Semi-Dry Transfer Cell (Bio-Rad). Membranes were probed with the antibodies against VDR (GR37 Calbiochem, VWR, Carnaxide, Portugal) and α-tubulin (T6199 Sigma Aldrich). Quenching and immunostaining of the blots was done according to the manufacturer's instructions. Detection was done using Amersham ECL Western Blotting Detection Reagents (GE Healthcare). Immunoblots were performed at least three times, and representative figures are shown. The BioRad Quantity One 1-D Analysis Software (Bio-Rad) was used to quantify the band intensities.

### Nucleic acid extractions

Cell lines were cultured in 75 cm^2 ^flasks to 70-80% confluency and harvested using 1 ml/10 cm^2 ^of TRIzol reagent (Invitrogen). Both RNA and DNA were extracted with TRIzol reagent according to the manufacturers' protocols. Nucleic acids were quantified using a NanoDrop ND-1000 (NanoDrop Technologies, Wilmington, USA) and quality was visually confirmed by gel electrophoresis.

### Microarray experiments

#### Arrays and data acquisition

For expression, NMD and CGH arrays hybridizations were performed onto slides containing 60-mer oligonucleotides representing 26845 unique genes based on The Human Release 2.0 oligonucleotide library as designed by Compugen (San Jose, California, USA), and obtained from Sigma-Genosys [15]. Following hybridization using a HybArray 12 (Perkin-Elmer, Zaventem, Belgium) the slides were scanned with a Microarray Scanner G2505B (Agilent Technologies Netherlands B.V., Amstelveen, The Netherlands). Spot analysis and quality control were fully automated using BlueFuse version 3.2 (BlueGnome, Cambridge, UK) and spots with quality flag <1 or confidence value < 0.1 were excluded from further analysis. Original data files for all arrays can be found in GEO (http://www.ncbi.nlm.nih.gov/geo/; accession numbers - GSE9867, GSE9887 and GSE9959).

#### Expression microarrays and analysis

A total of 14 microarray expression experiments were carried out, with four biological replicates and 10 technical replicates including dye swaps. cDNA probes were generated from 30 μg of total RNA, and experiments performed as previously described [16]. After scanning and data acquisition, genes with spots flagged in more than one array were excluded for further analysis. For the remaining genes, the value of a flagged spot was imputed using K nearest neighbors' imputation as implemented in the R-package "impute". Curvature in the MA plots was limited but nevertheless normalized using Lowess as implemented in the R-package "MAAnova" [17]. Using an analysis of variance (ANOVA) model, "dye", "hybridization date" and "sample" were specified as factors together with the factor of interest, "treatment" ("parental" minus "resistant"). MAAnova was used to perform ANOVA for all genes. Permutations were applied to the sample labels and uncorrected pooled *p*-values from the F-statistics were computed for the treatment effect. FDR corrected *p*-values were computed to adjust for multiple testing. PANTHER Classification System was used to statistically determine over- or under- representation of pathways http://www.pantherdb.org/[18].

#### Nonsense mediated decay arrays (NMD arrays) and analysis

Cells were incubated with the drug Emetine (Fluka), which inhibits the NMD pathway, hereby blocking the degradation of non-sense mRNAs [19]. Approximately 4 × 10^6 ^cells at 70%-80% confluence were treated with 100 μg/ml emetine for 10 hours [19]. Following incubation with emetine, cells were directly lyzed by the addition of TRIzol (Invitrogen) omitting any treatment with Actinomycin D. In total four hybridizations were performed (four biological replicates), one for each of the paired (MCF7 resistant vs. MCF7) emetine treated cell lines. For this reason the inclusion of a control experiments consisting of "normal" keratinocytes as suggested [19] was unnecessary and therefore omitted in the study. After scanning and data acquisition, spots flagged were excluded for further analysis and a distribution curve was built with the log_2 _ratios for the remaining genes. In typical conditions a normal distribution of the ratios is expected. By blocking the degradation of nonsense mRNAs an accumulation of genes with mutations is expected, which can be measured by a "skew" in the "normal" distribution plot towards the positive side.

#### Array comparative genomic hybridization (array CGH) and analysis

CGH arrays were performed as previously described [15]. DNA from resistant and sensitive cell lines were differentially labeled and co-hybridized. For interpretation and visualization purposes, smoothing was performed using "aCGH-Smooth" [20], with lambda set to 3.0. Smoothed log_2 _ratios of -0.15 and 0.15 were used as thresholds to define gains and losses.

### Quantitative PCR (Q-PCR) confirmation of siRNA and expression arrays

Gene expression was technically confirmed by Q-PCR using SYBR green chemistry. Non-redundant primers were designed and are listed in Table 1. Primer pairs were checked for linear response over a range of cDNA input and for nonspecific targets with dissociation curves. Total RNA (360 ng) was reverse-transcribed to cDNA according to the manufacturer's instructions (Multi-Scribe Reverse Transcriptase, Applied Biosystems, Nieuwerkerk aan den IJssel, The Netherlands) and subsequently used in the amplification with 10 μM gene specific primers and 12.5 μl of 2× SYBR green master mix (Applied Biosystems) in a total reaction volume of 25 μl. Reactions were carried out using standard cycle parameters on an ABI PRISM Sequence 7700 Detection System (Applied Biosystems). Relative transcript levels were determined using GAPDH as an internal reference. Statistically significant differences between resistant and sensitive cell lines were determined using comparative delta-delta Ct test [21]. All reactions were done in triplicate and expressed as mean of the values from three separate experiments.

### In silico analysis of array CGH profiles from solid breast tumors

Publicly available array CGH datasets were analyzed for the presence of alterations in the region 11q13.4-14.1. A total of four datasets corresponding to 356 individual profiles were screened [22-25]. The datasets were obtained from publicly available databases (GEO accession numbers: GSE8757 freeze March 2006, GSE6448 freeze May 2004 and ArrayExpress: E-TABM-170 freeze July 2003) and the journal website (http://www.pnas.org/content/99/20/12963/suppl/DC1, freeze October 2000). The 356 profiles included 315 breast tumors and 41 breast tumor cell lines. The CGHcall package [26] was used to identify alterations in the 13.5 Mb region at chromosome 11.

## Results

### Proliferation in 1,25D resistant and sensitive MCF7 cell lines

**Figure 1:**
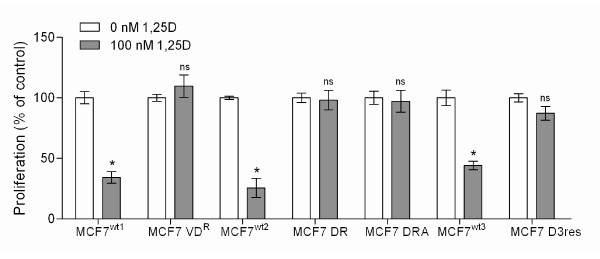
**Effect of 100 nM 1,25D or vehicle for 72 h on proliferation in the MCF7 cell lines used in the present study**. Data represents the mean ± SD of three values. Statistical significance was assessed by two-tailed unpaired Student's *t*-test. Differences were considered statistically significant when *p *< 0.01 (*). *p *> 0.01 was considered not significant (ns).

VDR is essential for regulation of calcium homeostasis by 1,25D, but its role in the anti-tumor effects mediated by vitamin D signaling remains unclear. Therefore, the objectives of these studies were first, to determine whether VDR knockdown would alter the anti-proliferative effects of 1,25D, and second, to identify genomic changes in stable 1,25D resistant cell lines. Studies were conducted with several MCF7 breast tumor cell lines previously selected for 1,25D resistance by independent laboratories [7,8]. We first confirmed the effects of 1,25D on growth of the three parental MCF7 breast tumor cell lines and the four variants selected for 1,25D resistance (Figure 1). In the presence of 100 nM 1,25D, proliferation was reduced approximately 50% in all parental MCF7 cell lines, according to the MTT assay. In contrast, 1,25D had no effect on proliferation in the resistant MCF7 cell lines. These data are in agreement with previous observations independently reported on these cell lines [7,8].

### Effect of VDR knockdown on 1,25D mediated proliferation inhibition

**Figure 2:**
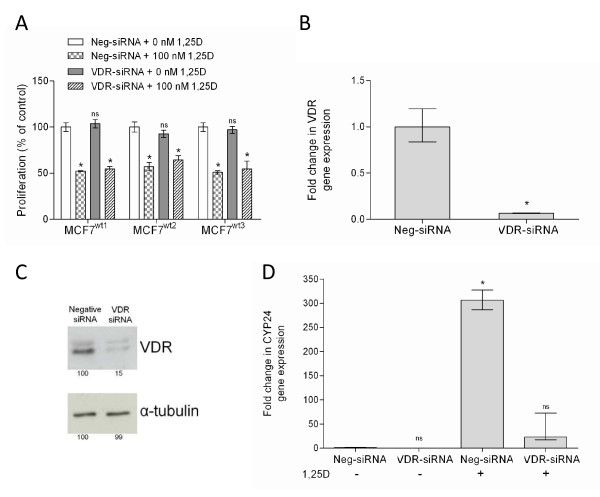
**Vitamin D receptor (VDR) knock down experiments through siRNA**. (A) MTT proliferation assay showing the effect of 100 nM 1,25D or vehicle after 72 h in cells transfected with a negative control siRNA (Neg-siRNA) and cells transfected with specific siRNA for VDR (VDR-siRNA). Results are expressed as % of control (Neg-siRNA transfected cells grown without exposure to 1,25D). (B) Q-PCR of *VDR *gene expression in cells transfected with neg-siRNA or VDR-siRNA normalized to GAPDH in the absence of 1,25D. (C) Western-blot for VDR and α-tubulin (loading control) in lysates from cells transfected with the negative siRNA or the VDR-siRNA. Band intensities are indicated below each lane. (D) Q-PCR of *CYP24A1 *gene expression in cells transfected with Neg-siRNA or VDR-siRNA. Data are mean ± SD of fold change in *CYP24A1 *expression in 1,25D treated cells vs. vehicle treated control. Statistical significance was assessed by two-tailed unpaired Student's *t*-test. Differences were considered statistically significant when *p *< 0.01 (*). *p *> 0.01 was considered not significant (ns).

Since VDR is pivotal in calcium metabolism we first tested whether VDR expression is correlated with proliferation and 1,25D sensitivity. For that reason, MCF7 cell lines were transfected with either a specific VDR-siRNA or a non-specific siRNA. The transfection in combination with the MTT proliferation assay allowed us to assess the effect of 1,25D on cells with reduced VDR expression. Surprisingly, parental MCF7 cells transfected with VDR-siRNA were growth inhibited by 100 nM 1,25D to the same magnitude as cells transfected with negative siRNA (Figure 2A). The degree of VDR knockdown by the VDR-siRNA was assessed in these cells at both the mRNA and protein level. By Q-PCR, a strong reduction of VDR transcripts was observed (Figure 2B and Additional file 1), and a comparable decrease in VDR protein expression was detected on western blots for VDR (Figure 2C and Additional file 1). Furthermore, we used Q-PCR to confirm that the VDR effector gene *CYP24A1 *was downregulated. In cells expressing negative siRNA, 100 nM 1,25D strongly (300-fold) induced *CYP24A1 *expression whereas in cells expressing VDR-siRNA this induction of *CYP24A1 *expression by 1,25D was not observed (Figure 2D). Besides being a downstream effector gene containing a VDRE, *CYP24A1 *is an important component of the 1,25D calcium metabolism pathway. Our data thus demonstrate that decreased VDR and *CYP24A1 *expression do not affect cellular sensitivity to the anti-proliferative effects of 1,25D. While unexpected, these data may indicate that a very low level of VDR expression is sufficient to trigger 1,25D mediated growth inhibition. Alternatively, 1,25D may mediate growth regulation in MCF7 cells via non-genomic processes [5,6,27].

### Identification of differential gene expression between 1,25D resistant and sensitive MCF7 cell lines

The siRNA results described above indicate that reduction of VDR expression did not necessarily de-sensitize cells to the anti-proliferative action of 1,25D. Consistent with the concept that VDR signaling can be dissociated from growth regulation, MCF7 cells selected for resistance to 1,25D retain VDR expression and function [7,8]. To assess the molecular mechanism that is involved in dictating cellular resistance to 1,25D, we performed genome wide analysis at the RNA and DNA level with these 1,25D resistant and sensitive MCF7 cell lines. These studies were performed in the absence of 1,25D in order to identify driver/causative alterations rather than 1,25D downstream genes involved in proliferation and cell cycle as described in the introduction. RNA isolated from all four 1,25D resistant cell lines was hybridized directly to their sensitive MCF7 counterparts. Both technical as well as biological replicates were combined in the analysis (in total 14 hybridizations). Using MAAnova, 1001 out of 26845 genes (3.7%) were differently expressed in 1,25D resistant MCF7 cells compared to the parental lines (see Additional file 2). The introduction of a Lowess normalization step had little to no impact on the overall results. When ranking the most significant differently expressed genes with respect to their log_2 _ratios, it was observed that no single gene was highly differentially expressed in relation to 1,25D resistance of all four cell lines. Instead, many genes had small increases or decreases in expression (Table 2 and Additional file 2). The differential expression of key genes was technically validated and confirmed by Q-PCR (Table 2), i. e. genes up-regulated in the array experiments are up-regulated in the Q-PCR, vice versa for down-regulated genes. Genes that were uniformly down regulated in the 1,25D resistant sub-lines included FAM14A, a poorly characterized interferon inducible gene; PHLDA1, a pleckstrin homology domain-containing protein involved in apoptosis; and CYP24A1, which codes for an enzyme that catabolizes 1,25D. Up-regulated genes included SLITRK6 an integral membrane protein generally expressed in neuronal cells; DDIT4, a stress response gene that initiates apoptosis; and CYFIP2, a gene involved in a redundant network of genes responsible for p53-dependent apoptosis. Down regulation of CYP24A1 in the resistant cell lines was surprising, as up-regulation of this gene has been linked to 1,25D resistance [28] and CYP24A1 was found to be amplified in various human tumors, including breast [29]. Consistent with previous reports [7,8], the VDR itself was not identified as differentially expressed in the resistant cell lines by these expression arrays. Because VDR was flagged in the array analysis due to low expression, we used Q-PCR analysis to confirm that VDR expression was not differentially expressed between resistant and sensitive cell lines.

**Table 2:** Selected list of genes associated with 1,25D resistance identified by expression array analysis^a^

Gene symbol	Accession Number [GenBank:]	Gene description	Chromosome location	Expression array (log_2 _ratio)	Q-PCR
Top 5 down-regulated					
FAM14A	NM_032036	Family with sequence similarity 14, member A	14q32.13	-2.03	-6.79
CYP24A1	NM_000782	Cytochrome P450 (Vitamin D 24-hydroxylase)	20q13	-1.20	-
PHLDA1	NM_007350	Pleckstrin homology-like domain, family A, member 1	12q15	-1.11	-3.69
ZFP276	NM_152287	Zinc finger protein 276	16q24.3	-1.02	-
-	BF244871	cDNA clone IMAGE:4083158	11	-0.98	-
Top 5 up-regulated					
SLITRK6	NM_032229	SLIT and NTRK-like family, member 6	13q31.1	0.98	-
BMP7	AK094784	Bone morphogenetic protein 7	20q13	0.96	-
-	BG115630	cDNA clone IMAGE:4417140	4	0.95	-
DDIT4	NM_019058	DNA-damage-inducible transcript 4	10pter-q26.12	0.91	2.64
CYFIP2	NM_001037332	Cytoplasmic FMR1 interacting protein 2	5q33.3	0.89	1.98
Additional^b^					
FOXC1	NM_001453	Forkhead box C1	6p25	-0.80	-2.05
CRABP2	NM_001878	Cellular retinoic acid binding protein 2	1q21.3	-0.66	-2.69
ST6GALNAC5	NM_030965	GalNAc alpha-2,6-sialyltransferase V	1p31.1	-0.10	-0.39
NUAK1	NM_014840	NUAK family, SNF1-like kinase, 1	12q23.3	0.75	2.66
VDR	NM_000376	Vitamin D receptor	12q13.11	-	0.13

-. Not available

^a^. Genes were identified considering FDR<0.05 as significant, with the exception of VDR due to flagging (see text for explanation)

^b^. Additional genes selected for Q-PCR quantification

### VDR signaling pathway is not identified by expression arrays on the 1,25D resistant cell lines

Since neither VDR nor CYP24A1 were implicated in the 1,25D resistance of these MCF7 cell lines, we examined whether other genes involved in the vitamin D pathway, or alternative signaling pathways, might be involved. To test this, we used pathway analysis to determine if the set of 1001 genes differentially expressed between the sensitive and resistant cell lines was enriched for genes serving particular pathways. Screening more than 160 pathways in PANTHER, six were found to be significantly altered in the 1,25D resistant cell lines relative to their parental counterparts. Notably, the VDR signaling pathway was not identified by this approach. Instead, five of the six identified pathways corresponded to membrane receptor signaling pathways involved in the cellular response to extracellular signals (Table 3): EGF, PDGF, FGF, Interleukins and Toll receptor pathways. This analysis also identified differential expression of genes in the B cell activation pathway in association with 1,25D resistance. Interestingly, these six signaling pathways were over-represented in the 1,25D resistant cell lines, and all are involved in the control of proliferation, cell growth and apoptosis. The specific genes in these pathways that were altered between sensitive and resistant cell lines are listed in Table 4. Additional experiments are needed to assess the involvement of these pathways in dictating cellular sensitivity to 1,25D.

**Table 3:** Pathways associated with 1,25D resistance identified using Panther Ontology gene enrichment analysis

Pathway	Over/under representation	FDR (<0.05)
B cell activation	+	0.01
EGF receptor signaling pathway	+	0.02
PDGF signaling pathway	+	0.02
Interleukin signaling pathway	+	0.03
FGF signaling pathway	+	0.04
Toll receptor signaling pathway	+	0.04

NOTE: + denotes over-representation in 1,25D resistant cells

**Table 4:** Genes present in at least three of the significant pathways identified

		Panther Ontology Pathway					
		
Gene symbol	Accession number [GenBank:]	B cell	EGFR	PDFG	Interleukin	FGF	Toll
PIK3CB	NM_006219	+	+	+	+	+	
GRB2	NM_002086	+	+	+	+	+	
PLCG2	NM_002661	+	+	+		+	
SOS1	NM_005633	+	+	+	+	+	
YSK4	NM_025052	+	+	+	+	+	
MAPK9	NM_139068	+	+	+		+	+
MAP2K3	NM_002756		+			+	+
STAT5B	NM_012448		+	+	+		
RASAL2	NM_170692		+	+		+	

NOTE: B cell activation; EGF receptor signaling pathway; PDGF signaling pathway; Interleukin signaling pathway; FGF signaling pathway; Toll receptor signaling pathway; + denotes involvement in the pathway.

### Nonsense mutations were not identified in 1,25D resistant cell lines

Nonsense mediated decay (NMD) analysis was performed to identify genes that might have undergone mutational events related to the 1,25D resistance phenotype. Resistant and sensitive cell lines were treated with emetine to block degradation of nonsense RNAs. Hybridizations were performed of resistant vs. sensitive emetine treated cell lines such that all 4 biological replicates were hybridized on a total of 4 arrays. The results indicated that no genes were recurrent in the biological replicates. In addition, the top positive log_2 _ratio was similar to the top negative log_2 _ratio. Visualization of all log_2 _ratios further revealed a normal distribution of the data around zero with no gene that stood out to the positive side of the curve. Thus, overall this data did not support the concept that the 1,25D resistant cell lines shared one or more common mutated genes.

### Resistant cell lines exhibit copy number reduction of chromosome 11q13.4-14.1

**Figure 3:**
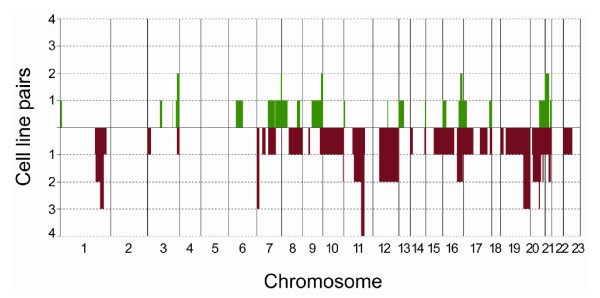
**Frequency plot for chromosomal alterations identified by array CGH with 1,25D resistant vs. sensitive MCF7 cell lines**. Alterations are ordered by chromosomal position and the y-axis indicates the number of cell line pairs in which the given alteration occurs. Green represents gains and red losses.

Chromosomal copy number aberrations (CNAs) affect the properties of a cancer in a (sub-) type specific manner [30]. Using array CGH, a variety of CNAs, including gains, losses and high level amplifications on multiple chromosomes were identified in MCF7 cell lines [31]. These chromosomal changes can occur spontaneously during *in vitro *culture or in response to environmental factors such as drugs [32]. Therefore, in the current study we were interested to determine whether *in vitro *selection for 1,25D resistance induced recurrent chromosomal alterations in MCF7 cells. To monitor chromosomal aberrations array CGH was performed. Direct hybridization of DNA from sensitive and resistant cell lines yielded primarily flat profiles, with few regions showing copy number alteration. As a control, the separate channels of these dual channel CGH arrays were interchanged using a common reference (across array) to verify the original MCF7 profiles [33]. Of those regions that were altered between sensitive and resistant cell lines, 76% showed reduction in copy number, but only one region was commonly altered in all four 1,25D resistant cell lines and none of the parental counterparts (Figure 3). The altered region varied in size with the smallest region of overlap (SRO) of 13.5 Mb and ranges from 11q13.4 to 11q14.1. The region contains 80 known genes [34], none of which are known to be involved in the vitamin D signaling pathway. We hypothesized that if loss of vitamin D responsiveness contributes to human malignancies, alterations in chromosome 11q13.4-14.1 might also be frequent in solid tumors. Publicly available array CGH datasets were used to screen for alterations at 11q13.4 to 11q14.1 in breast tumors. This *in silico *analysis showed that 40.6% of the breast tumors and 41% of the breast tumor cell lines exhibited alterations in this particular region of chromosome 11.

## Discussion

The molecular mechanisms underlying the anti-proliferative effects of 1,25D are poorly understood, despite the well known role of 1,25D in the regulation of calcium homeostasis. In this study we show that the anti-proliferative effects of 1,25D may be functional in MCF7 breast tumor cell lines, without a role for the central player in calcium homeostasis, VDR. These results were unanticipated since several known regulators of cell growth, such as *p21 *and *GADD45A*, contain functional VDREs in their promoter region and are induced by the 1,25D-VDR complex in cancer cells [35,36]. Furthermore, cell lines derived from VDR knockout (KO) mice are resistant to 1,25D mediated growth arrest [37], and re-introduction of VDR into these cells restores sensitivity to 1,25D mediated growth inhibition (Keith and Welsh, unpublished). While additional studies are needed to determine whether residual VDR activity in the MCF7 cells expressing VDR-siRNA is sufficient to mediate growth inhibitory signals upon binding 1,25D, our data demonstrates that marked reductions (~90% at the transcript level and ~85% at the protein level) in VDR do not necessarily induce 1,25D resistance. Seemingly contradictive to the role of VDR, this finding is consistent with previous data from MCF7 sub-lines selected *in vitro *for resistance to 1,25D, in which VDR function is retained [7,8], indicating dissociation between VDR expression and 1,25D mediated growth inhibition.

In the light of these results, we hypothesized that driver/causative genes other than VDR, or alternative pathways unrelated to vitamin D calcium signaling might impact 1,25D sensitivity or replace its function. Therefore we used genome wide screens to identify genetic alterations that cause resistance in our model. Indeed, in all four 1,25D resistant cell lines one common chromosomal region had reduced copy number at 11q13.4-14.1 (Figure 3). Eighty genes are present in this 13.5 Mb chromosomal region, none of which could readily be assigned to 1,25D mediated VDR signaling. In focally aberrant regions (<3 Mb) identification of driver genes is often straight forward, but it is our experience [38], acknowledged by others [39] that identification of candidate genes in larger chromosomal regions is problematic. One effort made was integration with our expression array data which did not proof helpful; hence pathway analysis of genes in this region would be arbitrary. Other authors performed similar array experiments in the presence of 1,25D, yet again none of the 80 genes in this region was identified [9-13]. Our expression array analysis confirmed that 1,25D resistant cells did not exhibit significant reductions in VDR gene expression. Still, as described previously the VDR-transcriptome may be significantly altered [12]. Indeed, these authors showed that *CYP24A1 *was the only induced gene that was common to the genetic profiles of the parental and the 1,25D resistant cell lines when treated with 1,25D treated [12]. In contrast, our expression array analysis identified *CYP24A1 *as one of the top five down-regulated genes in the 1,25D resistant MCF7 cell lines. Since *CYP24A1 *is strongly regulated by 1,25D in a negative feed-back, but no 1,25D was present in our test, any discussion on this apparent down-regulation remains speculative.

Pathway analysis of the expression arrays did not identify any other known vitamin D pathway genes either. Instead, over-representation of multiple membrane receptor signaling pathways known to be involved in the control of proliferation and apoptosis were associated with the 1,25D resistant phenotype. The five membrane-bound receptor signaling pathways identified in our analysis share the Grb2 and SOS genes and drive cell proliferation. Interestingly, previous analysis of one set of these sub-lines indicated that, in the absence of 1,25D, the doubling time of the MCF7 D3res cells was shorter than that of the MCF7 sensitive cells from which they were derived [8]. Furthermore, using proteomic approaches we previously identified several proteins in the MAPK-ERK-RAS mitogenic pathway that were differentially expressed in the MCF7 D3res cells relative to their sensitive MCF7 counterparts under basal conditions [9]. Although mechanisms have yet to be clarified, chronic up-regulation of these mitogenic pathways may selectively disable VDR signaling, leading to 1,25D resistance. In support of this idea, RAS activation has been shown to induce 1,25D resistance in keratinocytes via direct phosphorylation of VDR [40].

In a previous study we used the same genome wide tools to identify the mechanisms of Gemcitabine resistance [32]. Results were simple and straightforward to interpret; Gemcitabine had selected for tumor cells with high level amplifications, leading to high expression levels of genes within that amplicon which resulted in drug resistance [32]. Here a similar simple mechanism for 1,25D resistance was not identified. A possible explanation may be that most therapeutic drugs (e.g. Gemcitabine) are synthetic and 1,25D is generated endogenously. Consequently, malignant cells would need to develop resistance to the anti-proliferative effects of 1,25D in a very early stage of their development to allow tumor expansion. Accordingly, we hypothesized that many solid tumors would also contain alterations at 11q13.4-14.1. Indeed, screening of four datasets covering 358 individual array CGH profiles showed that the region in chromosome 11q13.4-14.1 is highly unstable strengthening this hypothesis.

## Conclusion

Evidence accumulates demonstrating an important role for Vitamin D not only in the treatment but also in the prevention of cancer, mainly breast cancer. Dozens of vitamin D analogues have been developed by pharmaceutical firms with the goal of dissociating the potentially toxic calcemic actions of 1,25D from its anti-tumor actions for use in cancer therapy. Because vitamin D analogs are currently in approximately 20 clinical trials [2], understanding the mechanisms by which 1,25D mediates growth inhibition, as well as the basis for development of 1,25D resistance, is crucial. Our genome wide screening analysis has identified chromosomal regions, membrane receptor pathways and new candidate genes outside of the classical VDR signaling pathway that may be associated with 1,25D resistance. These data provide new avenues for future explorations that may facilitate the development and use of vitamin D based drugs for cancer and other clinical applications.

## Authors' contributions

JLC carried out the experiments and analysis. PE assisted in the array experiments. MvdW supervised and assisted in all statistical analysis. CJN and JW established and provided three of the resistant cell lines. DtB conceived the VDR-siRNA experiments. JW, DtB and FS provided critical reviews of the manuscript. JLC and BY conceived and designed the study, and wrote the manuscript. All authors read and approved the final manuscript.

## Supplementary Material

Additional file 1 **SiRNA-VDR knockdown time course experiment**. The figure shows a time-course knockdown of VDR at 24 h, 48 h and 72 h both at the RNA (A) and protein level (B). Click here for file

Additional file 2 **Differentially expressed genes associated with 1,25D resistance**. The spreadsheet table contains a list with a detailed characterization of the genes associated with 1,25D resistance identified by expression arrays analysis considering a FDR <0.05 as significant. Click here for file
