# Supplementary figures and images for: Anti-proliferative action of vitamin D in MCF7 is still active after siRNA-VDR knock-down
Source: BMC Genomics. 2009 Oct 28;10:499. doi: 10.1186/1471-2164-10-499 (PMC2778664; doi:10.1186/1471-2164-10-499)

**A**

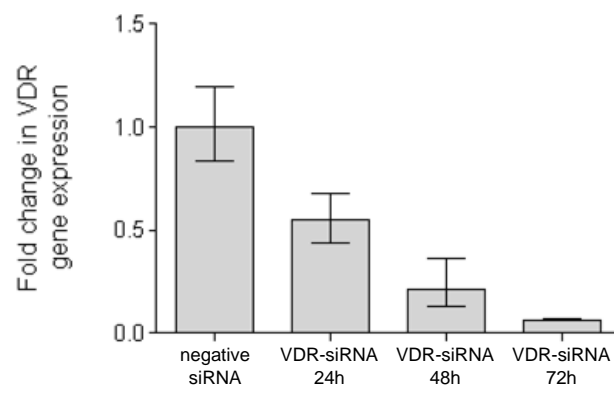

**B**

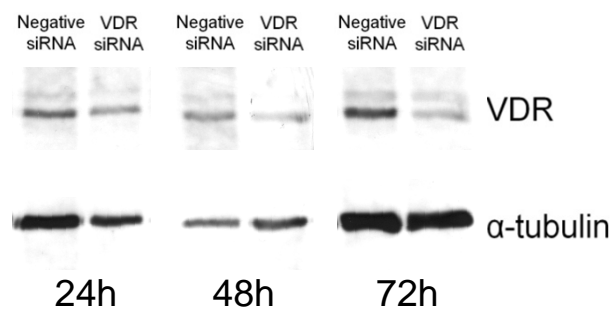

Supplement: Additional file 1 — SiRNA-VDR knockdown time course experiment. The figure shows a time-course knockdown of VDR at 24 h, 48 h and 72 h both at the RNA (A) and protein level (B). [file 1471-2164-10-499-S1.PDF]
